# Fever as an initial manifestation of spondyloarthritis: A retrospective study

**DOI:** 10.1371/journal.pone.0184323

**Published:** 2017-09-14

**Authors:** Se Jin Byun, William Han Bae, Seung Min Jung, Sang-Won Lee, Yong-Beom Park, Jason Jungsik Song

**Affiliations:** 1 Division of Rheumatology, Department of Internal Medicine, Yonsei University College of Medicine, Seoul, South Korea; 2 Institute for Immunology and Immunological Diseases, Yonsei University College of Medicine, Seoul, South Korea; University College London, UNITED KINGDOM

## Abstract

**Objectives:**

We aimed to evaluate a wide spectrum of clinical features of adult patients with spondyloarthritis (SpA) whose initial manifestation was fever, using the Assessment of SpondyloArthritis international Society (ASAS) classification criteria.

**Methods:**

We retrospectively collected the electronic medical records of hospitalized SpA patients who initially presented to the Severance Hospital (Seoul, Korea) with fever from January 2010 to May 2016. As a control group, we also recruited one-hundred consecutive patients who were diagnosed with SpA in our outpatient clinic. Clinical features and laboratory findings were compared in two patient groups.

**Results:**

There were 26 patients who had fever as initial presentation of SpA (reactive arthritis 50%, undifferentiated SpA 26.9%, ankylosing spondylitis 15.4%, enteropathic arthritis 3.8%, psoriatic arthritis 3.8%). Peripheral SpA was more common in febrile SpA patients than in control SpA patients (65.4% vs 24.0%, p<0.001). Febrile SpA patients were less frequently HLA-B27 positive than control SpA patients (52.2% vs 77.0%, p<0.05). At baseline, systemic inflammatory markers were significantly higher in the febrile SpA patients (white blood cell count, 11.57 vs 7.81 cells/μL, p<0.001; erythrocyte sedimentation rate, 69.2 vs 41.0 mm/h, p<0.001; C-reactive protein, 109.6 vs 15.3 mg/L, p<0.001). The proportion of patients treated with systemic steroids was significantly higher in febrile SpA patients (57.7% vs. 11.0%, p<0.001). The proportion of patients who visited rheumatology specialty was significantly lower in febrile SpA patients than in control SpA patients (7.7% vs 59.0%, p<0.001).

**Conclusion:**

Various subgroups of SpA can be presented with fever as an initial manifestation. Febrile SpA patients demonstrated higher systemic inflammation and a lower chance to visit rheumatology in early stage. When evaluating febrile patients with any clinical features of SpA, clinicians are advised to consider performing SpA-focused evaluation including HLA-B27 or a simple sacroiliac joint radiograph.

## Introduction

Spondyloarthritis (SpA) is a collective term to refer to a group of inflammatory joint diseases characterized by the distinguishing features of axial arthritis, asymmetric peripheral arthritis, dactylitis, and enthesitis. SpA subgroups are including ankylosing spondylitis (AS), psoriatic arthritis (PsA), reactive arthritis (ReA), undifferentiated SpA, and enteropathic arthritis [1]. According to a recently developed classification criteria by the Assessment of SpondyloArthritis international Society (ASAS), SpA is classified into axial SpA and peripheral SpA [2–4]. Axial SpA patients present characteristic symptoms of the axial skeleton (sacroiliac joints and spine), while peripheral SpA patients present symptoms of peripheral arthritis, enthesitis, and/or dactylitis [2,5].

SpA patients can be presented with a wide range of extra-articular symptoms such as skin rash, diarrhea, and eye discomfort [6]. Although fever has been known to be associated with certain subtypes of SpA such as ReA [7,8], fever as extra-articular manifestations of SpA has not fully been understood. A recent study showed that 33% of patients with ReA had fever when they came to hospital [8]. Although fever is not a common clinical feature of PsA or enteropathic arthritis, patients with generalized pustular psoriasis or inflammatory bowel diseases can be presented with fever during flare [9,10]. Recent findings suggest that although SpA has a wide range of clinical subtypes, they share fundamental disease pathogenesis [11] and there is a possibility that fever might be an extra-articular manifestation of SpA.

It is not that complicated to take care of febrile SpA patients in rheumatology clinic as long as they have the correct diagnosis. However, when undiagnosed SpA patients come to hospital with fever, it is not a straightforward case for primary care physicians or infectious disease specialists. In fact, SpA is difficult to diagnose when the patient’s main symptom is fever, often resulting in hospitalization for further diagnostic workups. SpA patients often remain undiagnosed for weeks or months, resulting in a significant delay in the appropriate treatments [12]. In the present study, we report a comprehensive evaluation of clinical features of SpA whose initial manifestation was fever, using ASAS classification criteria.

## Materials and methods

### Patient demographics

In the retrospective review of the electronic medical records in the Severance Hospital(Seoul, Korea) from January 2010 to May 2016, we identified that twenty-six patients were newly diagnosed with SpA while being admitted for the evaluation of undiagnosed fever (body temperature > 37.8°C) [13]. They were included in our study as febrile SpA patients. One hundred patients who were newly diagnosed with SpA at the outpatient clinic were included as control SpA patients. SpA patients with age <19 years were excluded. The medical records of SpA patients were identified using the following International Classification of Disease codes: M45.x (ankylosing spondylitis, AS), M07.39 (psoriatic arthritis, PsA), M00.99 (reactive arthritis, ReA), M06.00 (seronegative spondyloarthropathy), M02.39 (Reiter disease), M13.99 (enteropathic arthritis), H20.9 (anterior uveitis, AnU), M08.99 (juvenile idiopathic arthritis), and M48.99 (undifferentiated spondyloarthritis, uSpA). This study was approved by the Institutional Review Board of Severance Hospital (4-2016-0659). The requirement to obtain informed consent was waived because of the retrospective nature of the study.

### Classification of SpA subgroups

**Fig 1:**
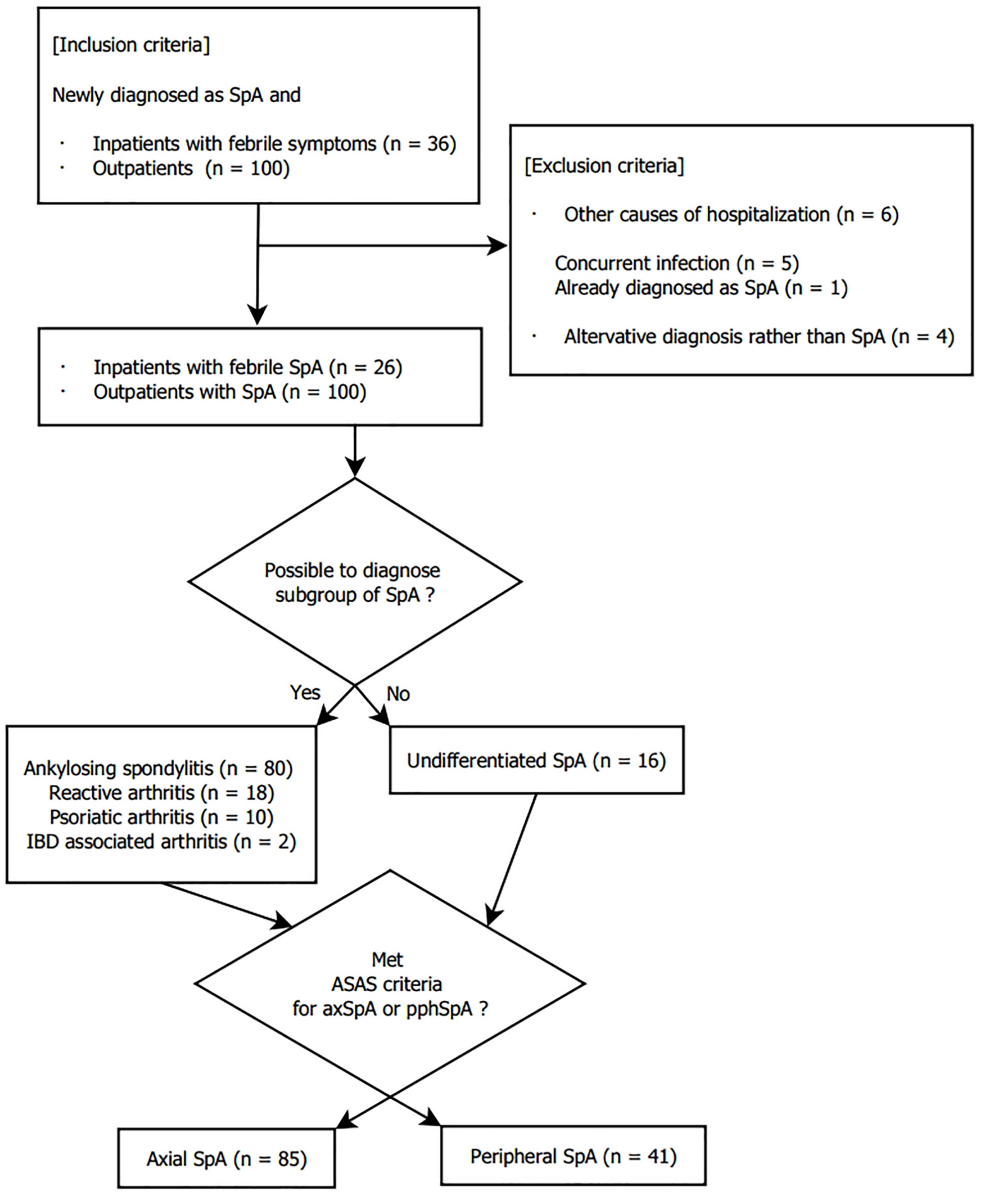
Flow chart of study enrollment. Abbreviations: SpA, spondyloarthritis; IBD, inflammatory bowel disease.

We reviewed the electronic medical records of the SpA patients to determine whether they meet the diagnostic criteria for SpA disease subgroups. We used the modified AS criteria for the diagnosis of AS [14], and the Classification Criteria for Psoriatic Arthritis criteria for PsA [15]. The diagnosis of ReA was made when the history of previous infections such as proven genitourinary or gastrointestinal infection, and typical musculoskeletal involvements were confirmed [16]. We excluded febrile SpA patients with evidence of active infections because it was not clear whether fever was the manifestation of infection or SpA. Enteropathic SpA were diagnosed when patients had inflammatory bowel disease with concurrent arthritis. When patients had several SpA features that did not fit into a single particular SpA subgroup, they were classified as undifferentiated SpA. All patients were classified into either axial or peripheral predominant SpA according to the ASAS classification criteria [2,3]. The flow chart of SpA classification is shown in Fig 1.

### Evaluation of clinical features

We reviewed the patients’ symptoms, physical findings, past medical history, and steroid use. Symptom duration was defined as time between the onset of dominant symptoms and the diagnosis of SpA. Symptoms can be eye discomfort, diarrhea, joint pain, back pain, skin rashes, or fever. In addition, laboratory findings, such as aspartate transaminase (AST), alanine transaminase (ALT), and C-reactive protein (CRP), erythrocyte sedimentation rate (ESR), and human leukocyte antigen (HLA)-B27 test, were evaluated at the time of diagnosis and at six months. Radiographic studies of the sacroiliac joint were reviewed. We used radiographic criterion based on modified New York criteria for AS which is sacroiliitis grade ≥2 bilaterally or sacroiliitis grade 3–4 unilaterally [14].

### Statistical analysis

We utilized the independent sample t-tests for normally distributed parameters, Mann–Whitney U test for non-normally distributed parameters, chi-squared test or Fisher’s exact test for categorical parameters, and Wilcoxon matched pairs test for comparison of clinical features. Statistical tests were two-sided. All statistical analyses were performed using IBM SPSS Statistics 22.0 software (IBM SPSS Inc., Chicago, IL, USA).

## Results

### Baseline demographics

Twenty-six SpA patients who initially presented with fever (febrile SpA) and one-hundred SpA patients diagnosed at the outpatient clinic (control SpA) were included. The mean ages of febrile SpA patients and control SpA patients were 39.6 and 36.7 years, respectively (Table 1). Male patients were 69.2% and 67.0% in each group. 7.7% of febrile SpA patients and 59.0% of control SpA patients received their initial subspecialty evaluation at the rheumatology clinic (p<0.001). The rest of febrile SpA patients were hospitalized by subspecialists in other departments such as emergency medicine (n = 16), infectious disease (n = 4), gastroenterology (n = 1), nephrology (n = 1), neurosurgery (n = 1), and orthopedics (n = 1). The mean duration of SpA symptoms was significantly seven-times shorter in febrile SpA patients than in control SpA patients (20.19±15.95 vs 140.19±196.89, p<0.001).

**Table 1:** Baseline characteristics of the study participants.

	Febrile SpA (*n* = 26)	Control SpA (*n* = 100)	p
Age, years	39.58±14.94	36.72±13.02	0.336
Male	18(69.2)	67(67.0)	0.829
Body mass index (kg/m^2^)	22.18±2.91	22.50±3.52	0.679
Duration of symptom, days	20.19±15.95	140.19±196.89	< 0.001
Direct referral to rheumatology	2 (7.7)	59(59.0)	< 0.001

Data are presented as mean±SD or n (%).

Abbreviations: SpA, spondyloarthritis

### Clinical features

We evaluated how many patients in each group had met the diagnostic criteria for SpA subgroups. AS was significantly less common in febrile SpA patients than in control SpA patients (15.4% vs 76.0%, p<0.001, Table 2). ReA was significantly more common in the febrile SpA patients (50.0% vs 5.0%, p<0.001). Febrile SpA patients had peripheral SpA more commonly than control SpA patients (65.4% vs 24.0%, p<0.001).

**Table 2:** Clinical features of febrile SpA patients.

	Febrile SpA (*n* = 26)	Control SpA (*n* = 100)	p
**Clinical subtypes**			
Ankylosing spondylitis	4 (15.4)	76 (76.0)	< 0.001
Psoriatic arthritis	1 (3.8)	9 (9.0)	0.686
Reactive arthritis	13 (50.0)	5 (5.0)	< 0.001
Enteropathic arthritis	1 (3.8)	1 (1.0)	0.371
Undifferentiated SpA	7 (26.9)	9 (9.0)	0.022
**ASAS classification criteria**			
Axial SpA	9 (34.6)	76 (76.0)	< 0.001
Peripheral SpA	17 (65.4)	24 (24.0)	< 0.001
**Peripheral joint involvement**			
Shoulder	7 (26.9)	16 (16.0)	0.158
Wrist & hand	3 (11.5)	25 (25.0)	0.103
Knee	12 (46.1)	21 (21.0)	0.023
Ankle & foot	5 (19.2)	15 (15.0)	0.359
**Clinical findings**			
Fever	26 (100.0)	1 (1.0)	< 0.001
Previous infection	13 (50.0)	6 (6.0)	< 0.001
Uveitis	3 (11.5)	16 (16.0)	0.571
Good response to NSAIDs	13 (50.0)	63 (63.0)	0.227
Arthroscopic surgery	3 (11.5)	0 (0.0)	0.008
HLA-B27	12 (52.2)*	77 (77.0)	0.016
Sacroiliitis in X-ray	12 (66.7)**	90 (90.0)	0.008

Data are presented as n (%).

*Number confined to patients who underwent each test (n = 23).

**Number confined to patients who underwent each test (n = 18).

Abbreviations: NSAIDS, non-steroidal anti-inflammatory drugs; SpA, spondyloarthritis

### Laboratory parameters

At the baseline, AST, ALT, CRP, and ESR were significantly higher, and hemoglobin and albumin were significantly lower in febrile SpA patients (Table 3). At six months, lab results were not significantly different between groups except CRP and hemoglobin.

**Table 3:** Comparison of laboratory parameters of febrile and control SpA patients.

	At baseline		p	At 6 months		p
	Febrile SpA (n = 26)	Control SpA (n = 100)		Febrile SpA (n = 26)	Control SpA (n = 100)	
White blood cell (cells/μL)	11.57±5.95	7.81±2.35	< 0.001	7.47±2.81	7.10±2.15	0.467
Hemoglobin (g/dL)	12.45±1.94	14.11±1.69	< 0.001	13.35±2.15	14.31±1.46	0.008
Platelet (×1,000/μL)	339.15±127.06	301.17±94.72	0.093	288.58±86	269.44±67.13	0.226
Total protein (mg/dL)	6.92±0.93	7.30±0.52	0.006	7.05±0.84	7.17±0.38	0.299
Albumin (mg/dL)	3.70±0.59	4.25±0.41	< 0.001	4.32±0.73	4.41±0.26	0.308
Creatinine (mg/dL)	0.86±0.49	0.75±0.16	0.083	1.00±0.96	0.78±0.17	0.033
AST (IU/L)	32.85±23.52	18.77±9.07	< 0.001	19.19±7.69	20.17±8.73	0.604
ALT (IU/L)	42.20±33.06	19.26±15.31	< 0.001	20.73±15.50	19.44±13.18	0.669
ESR (mm/h)	69.23±38.20	41.00±31.10	< 0.001	29.15±29.82	20.51±18.62	0.068
CRP (mg/L)	109.62±90.73	15.31±21.57	< 0.001	18.98±49.34	4.18±7.00	0.004

Data are presented as mean±SD.

Abbreviations: ALT, alanine transaminase; AST, aspartate transaminase; CRP, C-reactive protein; ESR, erythrocyte sedimentation rate; SpA, spondyloarthritis; TNF, tissue necrosis factor

### Treatment

The proportion of patients treated with systemic steroids was significantly higher in febrile SpA patients (at baseline, 57.7% vs 11.0%, p<0.001; after 6 months, 42.3% vs 22.0%, p = 0.046; Table 4). The proportion of patients treated with nonsteroidal anti-inflammatory drugs (NSAIDs) was significantly lower in febrile SpA patients (at baseline, 73.1% vs 95.0%, p = 0.003; after 6 months, 69.2% vs 96.0%, p<0.001). There were no significant differences in the proportions of patients taking tissue necrosis factor inhibitors and methotrexate in febrile and control SpA patients. At 6 months, the proportion of patients taking sulfasalazine was lower in febrile SpA patients than in control SpA patients (42.3% vs 67.0%, p = 0.025).

**Table 4:** Comparison of treatment between the febrile group and the outpatient group.

	At baseline		p	After 6 months		p
	Febrile SpA (n = 26)	Control SpA (n = 100)		Febrile SpA (n = 26)	Control SpA (n = 100)	
NSAIDs	19 (73.1)	95 (95.0)	0.003	18 (69.2)	96 (96.0)	< 0.001
Steroid	15 (57.7)	11 (11.0)	< 0.001	11 (42.3)	22 (22.0)	0.046
Sulfasalazine	6 (23.1)	56 (56.0)	0.071	11 (42.3)	67 (67.0)	0.025
Methotrexate	3 (11.5)	4 (4.0)	0.154	4 (15.4)	16 (16.0)	1.000
TNF inhibitors	0	0	N/A	5 (19.2)	11 (11.0)	0.320
Analgesics	4 (15.4)	6 (6.0)	0.213	5 (19.2)	8 (8.0)	0.140

Data are presented as n (%).

Abbreviations: N/A, not applicable; NSAIDS, non-steroidal anti-inflammatory drugs; SpA, spondyloarthritis; TNF, tumor necrosis factor

## Discussion

SpA has wide range clinical manifestations from axial and peripheral musculoskeltal symptoms to non-specific systemic symptoms. It is clinically challenging to make the diagnosis of SpA in patients with non-articular symptoms. A strikingly high number of SpA patients with non-specific symptoms are misdiagnosed by clinicians, resulting in inappropriate or delayed treatment [12,17]. According to a previous study, 50% of SpA patients with anterior uveitis were diagnosed with SpA after they developed the eye symptoms [18]. We found that only 7.7% of febrile SpA patients (Table 1) received their initial subspecialty evaluation with rheumatologists, suggesting that many clinicians do not consider SpA in the evaluation of febrile patients with extra-articular SpA symptoms. Furthermore, three febrile SpA patients (11.5%) underwent unnecessary arthroscopic surgery (Table 2), because the clinicians suspected septic arthritis in febrile SpA patients. However, we still lack a comprehensive understanding of clinical features of febrile SpA.

In the present study, we have retrospectively reviewed the electronic medical records of febrile hospitalized SpA patients. Febrile SpA patients were more likely to have peripheral joint symptoms, and higher white blood cell (WBC) count, CRP, ESR, AST and ALT, and lower total proteins, albumin, and hemoglobin than control SpA patients. Febrile SpA patients were significantly more frequently treated with steroids, compared to control SpA patients.

At six months, most of the laboratory abnormalities returned to normal except for hemoglobin, creatinine, and CRP. The proportion of patients taking steroids decreased in febrile SpA patients while the proportion of patients taking steroids increased in control SpA patients. It is probably because rheumatologists more easily decide to use steroid treatments when SpA patients have fever and systemic inflammation, while they are reluctant to use steroids on SpA patients with only joint pain in the early stage of treatment. However, after 6 months, when control SpA patients did not respond to first-line treatment (NSAIDs, sulfasalazine and methotrexate), the use of steroids might be increased. There were no significant differences in the proportions of patients taking tissue necrosis factor inhibitors in febrile and control SpA patients. This suggests that the prognoses of febrile SpA and control SpA patients are comparable. Our result is different from a previous observation in a pediatric population where febrile patients with enthesitis showed poor prognostic outcomes [19]. The discrepancy is likely due to the differences in the disease progression between child-onset enthesitis and adult-onset enthesitis.

In addition to conventional treatments such as NSAIDs and biologics, corticosteroid use in SpA patients has been evaluated in several studies. While local corticosteroid injections have been known to be an effective treatment in severe SpA [20,21], systemic use is not generally recommended in the treatment of SpA [22]. In this study, the proportion of patients taking systemic steroids in the febrile SpA group was higher than that in the control SpA group. Additionally, the use of NSAIDs, the first line therapy in SpA, is lower in febrile SpA patients probably related with higher use of steroids. Previous studies also suggested systemic steroid use in refractory peripheral SpA patients [23,24].

The physician’s suspicion appears to be important for the early diagnosis of the SpA [25]. AS is the best-known form of SpA with its characteristic chronic inflammation of spine. However, in febrile SpA patients, the relatively small proportion of AS makes it difficult for clinicians to make the proper diagnosis. Furthermore, peripheral SpA was significantly more common in febrile SpA patients. The clinical picture of febrile patients with knee pain and swelling can easily mislead clinicians to consider that the patients may have septic arthritis. Therefore, it requires special attention to detect SpA in febrile patients. Radiographic sacroiliitis was frequently detected (66.7%) in the febrile SpA group. 52.2% of the febrile SpA patients were positive for HLA-B27. A simple radiograph of the sacroiliac joints and the HLA-B27 test can be cost-effective screening tools in febrile patients with any clinical features of SpA.

There are limitations to this study. First, the data for this study were collected retrospectively, which may have led to bias in patient selection and analysis. Because it is not easy to make proper diagnosis of SpA in febrile patients, there is a possibility that our study population only represents a small portion of febrile SpA patients. Second, because this is an observational, cross-sectional study, the long-term prognostic outcomes were not evaluated. Third, there is a possibility that the difference between the febrile group and the control group could be associated with the disease severity because febrile SpA patients were treated as inpatient while the control group patients were treated as outpatient. This could cause selection bias because hospitalized patients were more thoroughly investigated including HLA-B27 tests. Fourth, we could not confirm that SpA is the direct cause of fever and there is a possibility of hidden concurrent infection. However, our patients had a thorough evaluation during hospitalization including infectious disease specialist consultation and there was no evidence of infection. They were treated with steroids which made fever and clinical features of SpA improved. After 6 months, 42.3% of febrile SpA patients were still taking steroids which demonstrated that our febrile SpA patients did have severe systemic inflammation which requires chronic use of steroids. These findings support that fever is associated with SpA. However, this is the first clinical study to comprehensively evaluate the clinical features of febrile SpA patients. When patients with fever have SpA-associated symptoms, SpA-focused evaluation such as the HLA-B27 test and a simple radiograph of the sacroiliac joint are recommended.

## Supporting information

S1 File The datasets for statistical analysis. (XLSX)
